# Supplementary material for: Are carbon emissions trading and green financial instruments synergistic? -Comprehensive quantitative research based on content analysis
Source: PLoS One. 2024 Mar 7;19(3):e0298601. doi: 10.1371/journal.pone.0298601 (PMC10919601; doi:10.1371/journal.pone.0298601)
Supplement: S1 Data — (ZIP) [file pone.0298601.s001.zip › Data/地级市Z_F工作报告.zip!/░▓╗╒/╗┤─╧╩╨/└┤╫╘╣1⁄2╓┌║┼í╛┬φ┐╦╩2╛▌═°í┐_2016.docx]

各位代表：
    现在，我代表市人民政府，向大会报告工作，请予审议，并请市政协委员和其他列席人员提出意见。
    **一、2015年工作和“十二五”发展回顾**
    过去的一年，全市人民在省委省政府和市委的坚强领导下，认真贯彻党的十八大、十八届三中、四中、五中全会和习近平总书记系列重要讲话精神，主动适应经济发展新常态，有效化解各种风险和挑战，统筹做好稳增长、调结构、促改革、抓招商、惠民生等工作，完成了年初确定的目标任务。初步核算，实现地区生产总值770.6亿元，增长2.8%；财政收入131亿元，增长4.1%；固定资产投资781.9亿元，增长3.5%；城镇、农村常住居民人均可支配收入分别增长6%、8%；城镇登记失业率控制在4%以内；PM10平均浓度下降20.6%，节能减排实现年度目标，全市呈现经济企稳向好、生态持续改善、社会和谐稳定的良好局面。
    （一）经济保持平稳增长。面对复杂严峻的宏观形势和煤炭价格下行的压力，坚持把稳增长作为首要任务。扶持实体经济稳增长。全面贯彻落实中央和省稳增长战略要求，强化精准发力、综合施策，出台促进经济持续健康发展、金融支持实体经济发展、促进中小微企业发展等系列政策，不断增强企业内生动力。建立精准帮扶工业企业机制，全面落实减税降费政策，为企业减负10.4亿元，解决了一批长期困扰企业发展的土地、规划等历史遗留问题。通过设立续贷“过桥”资金、创新实施新型“政银担”合作等举措，新增贷款74.3亿元，增长8.3%；实现直接融资345.7亿元，完成年度任务的116%。扩大有效投入稳增长。坚持经济工作项目化、项目工作责任化，落实“四督四保”要求，强化双月经济工作督查和重点项目调度，建立投资等主要经济指标预警机制。安徽大数据交易中心等项目加快筹建，国瑞药业二期等项目开工建设，中安煤化一体化、中国移动（安徽）数据中心等项目加快推进，富华工业园、朱集西矿、平电三期等项目相继竣工。全年亿元以上省重点项目开工43个、续建129个、竣工35个，工业、服务业分别完成投资319.9亿元、410.5亿元。强化招商引资稳增长。坚持把招商引资作为经济工作的重中之重，编制招商项目库，出台招商引资扶持政策和考核办法。建立每月“两通报两调度一督查”机制，压实工作责任，细化工作任务，开展精准招商。全年新签约千万元以上项目257个、总投资569.4亿元，分别增长30.4%、34.7%；引进内资608.2亿元，增长8.7%；外商直接投资2.1亿美元，增长3.5%，全市招商引资氛围日渐浓厚。
    （二）结构调整步伐加快。坚持煤与非煤“双轮”驱动，探索具有淮南特色的产业转型新路子。明晰“235”产业转型路径。结合淮南实际，着眼未来发展，出台培育主导产业促进高成长性产业发展实施意见。新型能源基地建设水平逐步提升，新型煤化工、现代装备制造、新一代信息技术等产业主导地位逐渐凸显，现代服务业、旅游文化、光电新能源、现代医药、绿色有机农产品加工等产业加速成长。产业结构不断优化。启动实施调转促“4106”行动计划。设立总规模50亿元的产业发展引导基金，全年投放8.1亿元，支持重点产业发展和重点项目建设，促进大数据、“互联网+”、云计算与三次产业融合。实现战略性新兴产业产值51.3亿元，完成高新技术产业增加值23亿元。第三产业占GDP比重提高3.7个百分点，非煤产业占规上工业增加值比重提高3.5个百分点。实施创新驱动发展。出台实施创新驱动发展战略加快创新型城市建设等3个文件，省级创新型城市建设试点深入推进。设立1亿元天使投资基金。全社会研发经费投入16亿元，占GDP比重达2.03%。授权发明专利483件，增长110%，万人发明专利拥有量3.97件。获批院士工作站3家、省级工程技术研究中心7家。10人入选省战略性新兴产业技术领军人才。新增“智慧企业”86家、高新技术企业13家。淮南（凤台）现代农业科技示范园跻身国家级园区，煤化工产品检验检测中心通过国家验收。
    （三）改革开放深入推进。坚持以改革破难题、以开放促发展。改革实现新突破。加快转变政府职能，强化简政放权、放管结合、优化服务，构建“五清单、一办法”权力运行监管体系，取消、冻结、转确认行政审批事项12项，政务服务满意度测评位列全省第一。进一步放开市场准入，建立政府核准投资项目目录。积极创新投融资机制，推广政府与社会资本合作模式，2个项目入选国家项目库。商事制度改革走在全省前列，“先照后证”、“三证合一”、“一照一码”和事中事后监管工作全面实施。新增各类市场主体16344户，新增“四上”企业139家。积极推广中国(上海)自由贸易试验区可复制改革试点经验，复制推广事项29项、借鉴推广6项。金融综合改革持续推进，新增上市企业和“新三板”挂牌企业各1家，在省区域性股权交易市场挂牌企业14家。机构改革持续推进，集中治理机关事业单位“吃空饷”问题，在全省率先创新推行公安辅警“六统一”改革。市级公务用车制度改革全面完成。国有企业改革稳步推进，组建市建设发展集团和市产业发展集团，成立淮南职业教育集团，启动市燃气总公司改革重组，淮南中北巴士公司股权回购有序推进。财税体制改革不断深化，市区财税管理机制和转移支付制度加快完善，盘活财政存量资金11.7亿元。全年完成改革任务73项。开放彰显新格局。与塞尔维亚武贝市结为友好城市，不断加强与友城间的交流合作。中美煤矿安全健康合作项目、中德城市智能交通系统项目落户淮南。淮南豆腐制作工艺亮相意大利米兰世博会。积极参与长三角经济协调与合作，加快与合肥经济圈城市融合发展。深化与央企合作，在建项目16个、竣工项目13个，累计完成投资642.2亿元。“百家民企进皖北——淮南行”活动成果丰硕。合肥海关淮南办事处获批挂牌运行。全年实现进出口3.3亿美元，新增外贸登记备案企业57家。
    （四）城市功能不断完善。围绕推进新型城镇化和全国智慧城市试点，加快提升城市功能和品质。丰富完善规划体系。坚持规划引领，注重“多规合一”，在科学编制“十三五”经济社会发展规划的基础上，编制完成合淮一体化规划、西部空间整合规划、淮南港总体规划，加快编制城市空间特色规划、淮河岸线资源总体规划，深入开展规划卫片图斑核查整改，为全市经济社会发展提供了有力支撑。大力推进城市建设。新城区建设提质提速，老城区改造步伐加快，西部城区环境整治有序推进。按照产城一体的要求，整合完善高新区与山南新区、经开区与大通区、煤化工园区与潘集区管理体制。全年棚改项目开工22个、共108万平方米，维修改造老旧小区65个，新改建各类地下管网200公里，建成城市绿道30公里。深入开展闲置土地清理处置、规范房地产市场等专项整治，土地和房地产市场秩序逐步向好。全年拆除各类违法建设301.2万平方米，集中整治城市私设道路障碍物447处，城市综合执法和管理水平进一步提升。加快发展综合交通。优化对外交通体系，商合杭高铁淮南段开工建设，济祁高速公路淮南段、3座淮河大桥、煤化工码头等建设加快，九龙大道等建成通车，新建、改造国省干线7条。城乡路网不断完善，广场路、淮舜南路下穿工程建成投用，林场路、朝阳东路、卧龙山路西延工程建成通车，农村公路建设三年行动计划全面实施，改造县乡道路80公里，新增（延伸）村道105公里。
    （五）“三农”基础进一步夯实。落实各项强农惠农政策，全年“三农”支出35.2亿元，增长2.9%。农业生产进一步发展。农业产业化步伐加快，新增农民专业合作社157家，市级以上龙头企业达151家，规上农产品加工企业达200家。农村土地确权工作基本完成。新增土地流转面积5.3万亩。积极推进沿淮洼地治理，小型农田水利设施改造提升、禹王泵站改造等工程完工。农村建设进一步加强。建成58个美好乡村中心村，启动7个美丽小镇建设试点。完成农村危房改造2730户、农房建设与改造2500户。解决8.6万农村居民和2.77万农村学校师生饮水安全问题。3个村镇荣获全国文明村镇称号。农民生活进一步改善。农村常住居民人均可支配收入达11391元。实施80个村扶贫开发整村推进，减少贫困人口8400人。惠农“一卡通”发放涉农补贴7.3亿元。务工人员工资保障和维权服务工作全面加强。
    （六）民生和各项事业持续发展。始终把民生作为一切工作的出发点和落脚点，圆满完成33项省级民生工程。全年13大类民生支出128.3亿元，占财政总支出的83.6%。高度重视就业工作。采取更加积极的就业创业政策，制定实施“创业淮南”三年行动计划。拓展就业新路径，城镇新增就业4.5万人，转移农村劳动力2.6万人，稳妥分流安置近万名关闭退出小煤矿职工。实施企业稳岗补贴政策，发放稳岗补贴1.27亿元，惠及23.4万职工。推进“工学一体”就业就学试点，对来淮就业创业高校毕业生实施“四补贴一扶持”。持续完善社保体系。完成各类参保324万人次，提高3.8%。新农合参合率达101.1%。提高最低工资标准，平均每月增加企业退休人员基本养老金191.1元、城乡居民基础养老金15元。统筹推进安居工程。全面完成省下达的保障性安居工程建设任务，开工12129套、基本建成15092套、入住6514户。开工沉陷区搬迁安置房4800套，搬迁入住4900户、1.23万人。积极推进货币化安置，满足群众安置需求。加快发展社会事业。加大财力倾斜，改善教学条件，提升教育质量，大通、田家庵、八公山3个区顺利通过国家义务教育发展基本均衡县（区）认定。新建幼儿园、中小学11所，市职教中心建成投用，安理大新校区加快推进。市一院门急诊医技楼建成投用，市四院病房楼基本建成。大力开展全民健身行动计划，建成45个农民体育健身等工程。推进文化旅游融合发展，开展“送戏进万村”等文化惠民活动6900余场，创建国家4A级旅游景区1处。人口自然增长率7.54‰。地震、气象、档案、民族、宗教和地方志工作不断加强，妇女、儿童、老龄和残疾人事业取得新的成效。

    （七）生态文明建设和社会治理体系加快完善。坚持安全发展、绿色发展理念，加快社会治理法治化进程。生态环境质量持续改善。国家森林城市、全国水生态文明试点市创建工作深入推进。扎实开展“千万亩森林增长工程”和“三线三边”绿化提升行动，完成成片造林1.95万亩。持续推进大气污染防治，完成7个重点工业企业除尘改造项目，整治混凝土搅拌站39个、煤场堆场630个、燃煤锅炉631个、非法码头62个，淘汰黄标车4015辆，PM10平均浓度降幅全省第一。积极开展水污染综合防治，焦岗湖国家湿地公园通过国家验收，淮河淮南段水质稳步改善。采煤沉陷区综合治理世行贷款项目正式获批。社会治理体系不断完善。强化安全生产监管，在食品药品、工业生产、交通运输、公共安全等领域开展系列专项整治。深化平安淮南建设，打击各类违法犯罪活动成效显著，数字化立体化治安防控体系加快构建。市长热线办理群众来电8万余件，回复网民留言1400余条。社会服务管理信息化建设不断加强。加大定期接访、带案下访力度，及时解决群众反映强烈的突出问题。国防教育、国防动员和人民防空工作扎实推进，军民融合发展取得新成效。

    各位代表！2015年，我们实现了“十二五”的圆满收官，也为“十三五”的顺利开局奠定了坚实基础。回首过去五年，我们紧紧围绕“推动转型发展、建设美好淮南”的宏伟目标，坚定信心，保持定力，妥善应对各种挑战，有效化解诸多矛盾，积极抢抓发展机遇，带领全市人民团结奋进、苦干实干，实现了经济社会和各项事业的新发展。五年来，经济实力不断增强。地区生产总值由2010年的604.2亿元提升到2015年的770.6亿元，年均增长7.3%。财政收入由2010年106.1亿元提升到2015年的131亿元，年均增长4.3%。粮食生产实现“十二连增”。产业结构不断优化。煤电产业长足发展，非煤产业不断壮大，战新产业加速培育。三次产业结构比为9:51.6:39.4，现代服务业占GDP比重提高11.6个百分点，非煤产业占比提高2.7个百分点。发展活力不断释放。行政管理体制、要素市场体系日趋完善，累计新增市场主体75966户。对外交流不断深化，区域合作更加紧密，园区平台加快升级。城乡面貌不断改善。老城区改造力度加大，新城区建设步伐加快。智慧城市建设正式启动，美好乡村建设成果丰硕，城乡生活环境明显提升。全市建成区面积115平方公里，常住人口城镇化率68.2%。生态环境不断优化。节能减排任务超额完成，大气环境质量明显改善，水环境治理取得积极成效。严守耕地保护红线，连续14年实现占补平衡。国家园林城市、全国绿化模范城市等创建成果持续巩固。民生福祉不断增强。城乡居民收入保持较快增长，收入增速明显高于GDP增速。五年投入民生资金581.9亿元。新增城镇就业26.9万人。社会保障体系逐步健全。文化旅游强市战略深入实施。各项事业加快发展。社会治理体系日趋完善。依法行政能力不断提升。深入实施《全面推进依法行政实施纲要》，自觉接受人大法律监督和政协民主监督，办理人大代表议案建议738件、政协提案2009件，提请审议地方性法规11件，制定政府规章10件。坚持科学民主依法决策，制度建设质量显著提升，权力清单和责任清单编制完成并公布运行，政务公开更加规范，行政监察和审计监督体系进一步完善。“三严三实”专题教育扎实开展，廉政建设和反腐败力度不断加大，一批违法违纪行为得到严惩，风清气正、干事创业的政治生态进一步形成。

    各位代表！2016年伊始，我们迎来了行政区划的重大调整，淮南的发展翻开了崭新的一页。“十二五”期间，寿县县委县政府团结带领全县人民，推动经济、政治、文化、社会、生态“五位一体”发展，地区生产总值达133亿元，财政收入由3.9亿元跨越到10.7亿元，城乡居民人均可支配收入分别达19314元、8594元，贫困人口由22.26万减少到9.01万，综合实力实现新提升，城乡建设展示新风貌，人民生活得到新改善，各项工作迈上新台阶。我们坚信，在市委市政府的坚强领导下，在全市上下的大力支持下，寿县人民一定能够开创更加美好的未来。

    在肯定成绩的同时，我们也清醒地认识到，当前的政府工作与新形势、新要求还有一定差距。宏观经济环境错综复杂，经济社会发展中不协调、不平衡因素增多，抗风险和应对挑战能力仍需提升；脱贫攻坚任务艰巨，群众生活水平仍需提高；环境治理任重道远，影响稳定的因素依然存在；干事创业氛围不浓，作风建设仍需加强。对此，我们将牢记使命、勇于担当，采取切实措施，认真加以解决。

    回首不平凡的五年，我们深刻体会到，做好新形势下的淮南工作，必须把中央精神、省里要求和淮南实际结合起来，努力走资源型城市可持续发展之路；必须扭住发展第一要务不动摇，坚持一张蓝图干到底，调结构、转方式、促升级，打基础、管长远、增后劲；必须推进体制机制创新，以改革破解难题、以创新释放动能、以开放激发活力，使要素配置更加高效、创新创业氛围更加浓厚；必须坚持统筹协调发展，促进城乡建设一体化、公共服务均等化、群众利益最大化，不断提升群众的幸福指数；必须加快政府职能转变，坚持依法行政，践行“三严三实”，凝聚加快发展的强大合力。

    各位代表！经验弥足珍贵，成绩来之不易。这是市委坚强领导、市人大依法监督、市政协民主监督的结果，也是全市广大干部群众同心协力、奋力拼搏的结果。在此，我代表市人民政府，向全市人民，向驻淮部队、武警官兵和政法干警，向各民主党派、各人民团体和各界人士，向关心支持淮南的同志们、朋友们，向在淮的投资者、建设者和创业者，表示衷心的感谢和崇高的敬意!

**二、“十三五”奋斗目标和主要任务**

    “十三五”是全面建成小康社会的决胜期，也是加快转型发展的战略机遇期，做好这一时期的工作尤为重要。今后五年的指导思想是：高举中国特色社会主义伟大旗帜，全面贯彻党的十八大和十八届三中、四中、五中全会精神，以马克思列宁主义、毛泽东思想、邓小平理论、“三个代表”重要思想、科学发展观为指导，深入贯彻习近平总书记系列重要讲话精神，坚持“四个全面”战略布局，坚持创新发展、协调发展、绿色发展、开放发展、共享发展，以提高发展质量和效益为中心，以加快调结构转方式促升级为主抓手，以增进人民福祉、促进人的全面发展为出发点和落脚点，持续实行“1235”发展思路、调转促“4106”行动计划，努力建成国家新型能源基地、国家资源型城市可持续发展示范区、国家创新型城市和全国重要的文化旅游目的地，确保如期全面建成小康社会，奋力开创美丽淮南建设新局面。

    今后五年的奋斗目标是：紧紧围绕如期全面建成小康社会，努力实现以下新目标。

    ——经济保持中高速增长。到2020年，地区生产总值和城乡常住居民人均可支配收入比2010年翻一番，居民收入增长和经济增长同步。人均指标在全省位次逐步提升。

    ——产业结构更趋优化。三次产业结构不断优化，服务业占GDP比重达41%，非煤产业占比明显提高。

    ——改革开放取得突破。重点领域和关键环节改革取得决定性成果。到2020年，进出口总额达5亿美元，年均增长6%。

    ——文明程度不断提高。中国梦和社会主义核心价值观更加深入人心。现代公共文化服务体系基本建成。新增就业劳动力受教育年限13.5年。社会治理体系更加健全。

    ——生态环境明显改善。PM10平均浓度下降13%，单位生产总值能耗、主要污染物减排完成省下达的目标，土壤环境质量达标率达85%，森林覆盖率逐年提高。

    ——民生保障显著提升。人民生活水平普遍提高。城乡居民就业稳定。12.26万贫困人口全部实现脱贫。户籍人口城镇化率提升到50%。

    ——制度体系更加健全。体制机制加快完善，法治政府基本建成，司法公信力明显提高，全社会法治意识不断增强。区域协同发展机制基本建立，各领域合作不断深化。

    实现上述目标，必须把中央“创新、协调、绿色、开放、共享”发展理念，与淮南转型发展的实际有机结合起来，唱响全面转型主旋律，让淮南转型之路越走越宽、越走越好。

    （一）把创新发展作为全面转型的战略引擎。树立抓创新就是抓发展、谋创新就是谋未来的理念，加快形成以创新为引领和支撑的发展模式，促进经济迈向中高速、产业迈向中高端。深入推进战略性新兴产业集聚发展，加快培育3大主导产业、5大高成长性产业，打造在全省有重要影响力的战略性新兴产业集聚发展基地。深入推进传统产业信息化建设，落实《中国制造2025·安徽篇》行动计划，促进工业化和信息化融合发展，促进服务业优质高效发展，促进农业绿色、集约、安全、可持续发展，加快“淮南产品”向“淮南品牌”转变、向“中国品牌”升级。深入实施创新驱动发展战略，打造一批高新技术企业、科技小巨人企业，支持民营企业转型升级、提质增效、做大做强。构建发展新体制，深化行政管理体制、国资国企、财税体制、金融体制和农村综合等改革，推动淮南发展向创新型、效益型转变。

    （二）把协调发展作为全面转型的重要取向。遵循“一尊重、五统筹”理念，坚持规划先行，提高发展的协调性、平衡性和可持续性。推动区域协调发展，加强老城区改造，加快新城区开发，划定城市周边基本农田，防止城市建设“摊大饼式”四面扩张。推动产城融合，打造高新技术开发区与山南新区、淮南经济技术开发区与大通区、煤化工园区与潘集区、寿县新桥国际产业园与周边区域、凤台经济开发区与河东片区五大产城融合平台。推动县区经济振兴，加快园区特色发展，县区生产总值与全市同步增长，园区主要指标增长快于全市平均水平。推动市矿统筹发展，创新市矿协调发展机制。推动城乡一体化发展，构建“三环四通道”综合交通体系，推进新型城镇化试点，实现美丽乡村建设全覆盖。推动“六城同创”，形成物质文明和精神文明良性互动、协调共进的新格局。

    （三）把绿色发展作为全面转型的基本要求。坚持算长远账、算综合账，筑牢绿色底线，留足发展空间。健全生态文明制度体系，划定并严守资源环境生态红线。优化城市生态功能布局，落实主体功能区战略，支持寿县建设绿色发展试点县。实行最严格的环境保护制度，加大环境治理和执法力度，年均优良天气天数达322天以上、淮河淮南段水质优良比例达100%，主城区黑臭水体控制在10%以内。推动低碳循环发展，构建循环经济产业体系。实行能源和水资源消耗、建设用地等总量和强度“双控行动”，促进资源节约和高效使用。创新采煤沉陷区综合治理模式，提升综合治理水平。推进八公山、瓦埠湖、淠河等绿色生态建设。

    （四）把开放发展作为全面转型的活力源泉。坚持以开放促转型、以转型促开放，构筑开放型经济新优势。积极融入国家开放大格局，打造长三角地区能源供应基地及化工原材料基地，推动经开区创建国家产城融合示范区、高新区（山南新区）创建国家级园区、煤化工基地创建国家级基地。加快合淮一体进程，在合肥都市圈升级进程中发挥重要作用。推动淮河流域综合治理和振兴发展，优化沿淮产业和城镇布局。围绕主导产业、重点区域招商，促进引资引技引智一体化，引进内资、外商直接投资年均分别增长15%、8%。

    （五）把共享发展作为全面转型的根本主旨。按照人人参与、人人尽力、人人享有的要求，增强发展的溢出效应，让群众有更强的幸福感。持续增加居民收入，力争城乡常住居民人均可支配收入年均分别增长8.5%、10%，分别突破3.9万元、1.6万元。落实大别山集中连片扶贫攻坚规划，推进精准扶贫、精准脱贫。落实国家和省帮扶政策，坚决打赢脱贫攻坚战。坚持就业优先战略，城镇登记失业率控制在4.5%以内。坚持普惠性、保基本、均等化、可持续方向，统筹发展文化、教育、医疗、卫生等社会事业，扩大公共服务供给。逐步完善社会保障体系，基本实现法定人员全覆盖。依法加强和改善人口管理服务，保障妇女和未成年人权益，开展应对人口老龄化行动。完善权利公平、机会公平、规则公平的制度体系，让群众生活更美好、城市更包容、社会更温暖。

**三、2016年主要工作**

    今年是全面建成小康社会决胜阶段的开局之年，是推进结构性改革的攻坚之年。我们要立足当前，着眼长远，确保实现“十三五”发展良好开局。经济社会发展的主要预期目标是：地区生产总值增长6%，财政收入增长4.5%以上，固定资产投资增长10%，社会消费品零售总额增长9%，城镇和农村常住居民人均可支配收入分别增长6%、7%，城镇登记失业率控制在4.5%以内，节能减排完成省下达的目标任务。

工作中，重点抓好以下七个方面:

    （一）落实供给侧结构性改革，促进经济平稳增长

    在注重关注需求的同时，下大力气落实供给侧结构性改革举措，抓好去产能、去库存、去杠杆、降成本、补短板等重点任务，努力实现有质量、有效益、可持续的增长。

    积极化解过剩产能。严格环保、能耗、技术标准，推动煤炭等行业化解产能过剩。按照企业主体、政府推动、市场主导、依法处置的原则，妥善处置资不抵债、扭亏无望的“僵尸”企业。落实不良资产处置、失业人员再就业和生活保障、财政专项奖补等支持政策，强化政策托底功能，尽可能多兼并重组、少破产清算，确保经济运行有序。

    放开搞活实体经济。开展降本增效专项行动，落实结构性减税等政策，降低企业税费负担、财务成本、物流成本和融资成本。进一步清理规范中介服务，降低制度性交易成本，放权给市场、让利给企业、让主体多起来。推进直购电试点改革，降低企业用电成本。加大精准联系帮扶重点企业力度，帮助企业解决实际困难。实施民营经济提升工程，新增民营企业4000家以上、个体工商户1万家以上。

    全力抓好项目投资。把项目建设作为扩大投入的有效载体，加强重点项目调度，确保全年完成投资1000亿元以上，其中工业投资355亿元。实施项目建设年活动，全力推进中安煤化一体化、煤制天然气、光伏发电等重点项目，确保中国移动（安徽）数据中心一期、淮南新能源研究中心一期等项目竣工投产。积极对接国家和省重大规划和工程包，提高项目谋划的有效性和精准性。发挥政府投资的撬动作用，执行和示范一批政府与社会资本合作项目，进一步释放民间投资潜力。

    促进房地产市场健康发展。以满足新市民住房需求为重点，鼓励农民进城购房，引导城乡居民住房消费。深化住房制度改革，建立租售并举的住房保障制度。加大公租房分配入住和消化力度，逐步扩大公租房和住房公积金覆盖面。创新货币化安置方式，加快搬迁入住进程。加强房地产领域专项整治，依法依规处理各种违法违规行为，规范房地产市场秩序。

    加大招商引资力度。编制完成主导产业及高成长性产业招商行动计划，坚持领导招商、精准招商，建立招商专员、招商顾问制度。深化与央企、民企合作，积极引进全国500强、行业500强来淮设立地区性总部或分支机构。推进“三互”大通关建设，推动设立淮南商检正式机构。推进招商项目审批服务“交钥匙”工程，实行外资项目普遍备案、有限核准等管理制度。创新招商引资考核机制，完善优化发展环境责任追究办法，确保引进内资、外商直接投资、进出口稳步增长。

    （二）主攻产业转型升级，努力提高发展质量

    落实资源型城市转型发展系列政策，深入实施调转促“4106”行动计划，加快培育新的经济增长点。

    优化工业产业结构。坚持把工业作为转型升级的主战场，培育壮大新兴产业，改造提升传统产业。全年新增规上工业企业80户，规上工业增加值增长5%。支持煤炭企业多元化发展，推进潘集选煤厂建设，做好朱集西低热值煤电厂等前期工作，促进煤电联营。围绕“235”产业转型路径，加快大数据、云计算、“互联网+”、现代煤化工、生物医药等产业培育，打造战略性新兴产业集聚发展基地。实施新一轮技术改造和节能改造，完成技改投资220亿元。研究制定园区差异化发展办法，完善园区管理服务体制机制。

    大力发展现代农业。树立大农业、大食物观念，推动粮经饲统筹、农林牧渔结合、种养加一体，促进一二三产业融合发展。全面落实粮食安全责任制，开展粮食绿色增产模式攻关，粮食总产达260万吨。加强“菜篮子”工程建设，全年蔬菜播种面积72万亩。创新农业产业化合作模式和营销方式，发展“互联网+农产品加工”，支持淮南牛肉汤等地方特色产品做大做强。加强省级农业产业化示范区建设，创建省级现代农业示范区2个。培育新型农业经营体系，新增农民专业合作社155家、家庭农场75家。加强新技术、新品种、新模式创新，完善农业科技、疫病防控、农资配送、农机作业等服务，构建全程社会化服务体系。建设淠河生态经济带，打造农产品生产供应基地。加强农田水利基本建设，开工建设正硖段行洪区调整改造、淮干一般堤防加固工程，推进西淝河洼地治理，提升淮西湖、十涧湖综合排涝能力，完成世行贷款重点平原洼地治理项目，改善农业生产条件。

    促进服务业量增质优。开展改善消费品供给专项行动，优化消费环境，培育新兴消费。推进现代服务业集聚区建设，建成一批专业市场和特色商圈、街区。大力发展创意经济，引进研发中心、文化创意等高端服务业，打造3个文化创意园区。大力发展科技创新服务业，推进“一园一谷一中心”建设。积极发展现代金融业，支持企业上市融资，扩大新型“政银担”、“税融通”等业务。加快电子商务发展，布局农村电商生态圈，打造一批淘宝镇、淘宝村。加快发展现代物流业，建立完善以淮南新港、淮南无水港为依托的物流体系。发展生活性服务业，拓展家政服务、养老托幼、医疗健康、体育健身、实物租赁等服务新领域。

    （三）统筹城乡建设管理，完善宜居宜业功能

    围绕新型城镇化试点，统筹推进城乡规划、建设和管理，塑造美丽淮南新形象。

    强化规划引领。推进“多规合一”，构建“一个空间、一张蓝图”规划体系。启动城市总体规划、土地利用总体规划和八公山风景区总体规划修编，研究淮河以南城区全域城镇化规划。开展城市紫线、防洪和综合管廊等专项规划编制。完善农村人居环境、省级美丽乡村等村镇规划体系。依法加强规划管控，强化规划的严肃性和约束性。

    完善交通体系。大力发展立体交通、综合运输，有序解决过山、过河、过境等交通瓶颈问题。完善对外交通体系，启动江淮运河、西淝河复航等重点工程，加快商合杭高铁淮南段及3个站点、济祁高速公路淮南段建设，建成孔李淮河大桥、凤台淮河二桥，推进淮上淮河大桥建设。完善市政路网，开工建设东西部第二通道东延、中兴路等工程，建成朝阳路西延等工程，整修改造沿矿南路等11条道路。推进区域路网建设，加快县区间互联互通。完善交通设施，启动城市公共自行车系统、中心城区停车场和充电设施建设，更新公交车300辆，开通寿县至西部城区公交。

    提升城市功能。加快老城区改造，制定棚改及配套设施建设三年行动计划，全年开工保障性安居工程4896套、基本建成5218套，推进上郑广场、安成次中心、大通中心城区改造等项目建设，完成34个老旧小区维修改造。加快山南新区建设，推动光电产业园、盖天力医药产业园、淮南互联网经济产业园建设，在产城一体上取得新突破。开工万达广场、红星美凯龙、奥体宾馆等项目，推进台湾风情商贸文化城、绿沃城市综合体、淮矿新村、玉兰大道等项目建设，建成周集大坝公园、奥体中心主体场馆、春分街下穿隧道等项目。完善城市基础设施，加强水电气管网和地下管廊建设，启动潘集自来水厂扩容工程，推进3座泵站及配套管网、市第一污水处理厂扩建工程，加快城市公厕、菜市场、街头游园、城市绿道等配套设施建设，为群众生产生活创造一个满意的环境。

    加强城市管理。实施数字城管系统扩容升级,推广智能化城市公共管理模式。推进建筑垃圾、餐厨垃圾资源化利用，建成生活垃圾中转站10座、寿县生活垃圾焚烧发电项目。开展住宅小区物业管理、“五小行业”、户外广告、占道经营等专项整治。坚决拆除各类违法建设。启动城管体制改革，建立健全事权划分体系、经费投入体系和管理考核体系。进一步提升舜耕山风景区建设水平。推进“三治三增三提升”行动，强化县城规划建设管理。推进西部城区环境整治工程。加强寿县古城、古镇、古建、古址保护与利用。

    建设美丽乡村。推动美丽乡村建设“以点为主”向“由点到面”战略转换，建成27个美丽乡镇、51个省级美丽乡村中心村。改造农村危房1233户。实施农村道路畅通工程，改造县乡道路160公里、村道200公里、农村危桥13座。新改建农村变电站3座、线路177公里。实施农村安全饮水巩固提升工程，重点解决贫困村饮水安全问题。推进农村环境综合整治，实施城乡环卫一体化。

    （四）推进文化传承创新，打造文化旅游强市

    立足淮南人文底蕴和自然山水，整合文化旅游资源，促进融合发展，提升淮南的软实力和美誉度。

    加强精神文明创建。全面启动“六城同创”，深化群众性精神文明创建活动，实施文明城市连片创建，开展文明村镇和小城镇创建。推进思想道德、社会公德、职业道德、家庭美德、个人品德建设，开展淮南好人事迹宣传活动。加强网上思想文化阵地建设。广泛开展志愿服务，建立常态化机制。加强信用社会建设，构建政务诚信、商务诚信、社会诚信和司法公信体系。

    完善公共文化服务体系。推进文化惠民工程，加快安徽楚文化博物馆、农民文化乐园省级试点、乡镇综合文化服务中心建设，建成市文化艺术中心，做好公共文化馆免费开放工作。加快省级文化产业示范基地建设，推动中国淮南少儿艺术文化园、豆腐文化产业园等建设，文化产业增加值增长10%以上。加强文物、非物质文化遗产保护，支持寿县古城墙争创世界文化遗产、安丰塘争创全球重要农业文化遗产。繁荣发展哲学社会科学、文学艺术、新闻出版和广播影视事业。

    加快文化旅游融合发展。出台促进旅游业改革发展的实施意见，打造全国重要的文化旅游目的地。加强焦岗湖国家湿地公园等景区景点建设，支持寿县古城—八公山创建国家5A级旅游景区。加强旅游商品开发，打响八公山豆制品、紫金砚等特色文化旅游品牌。完善市级层面协调调度机制，统筹推进旅游规划、资源整合、市场推介等工作，提升综合服务功能。集聚旅游发展要素，启动寿县古城游客中心建设，开通双休日旅游直通车，力争全市旅游人次增长12%、旅游收入增长13%。

    （五）推进绿色低碳发展，提升生态文明水平

    坚持绿色富市、绿色惠民，建立严格的环境监管网格化管理、环保准入制度，提升自然生态系统稳定性和服务功能。

    全面加强大气环境治理。出台大气污染防治工作考核细则，巩固大气污染防治成果，确保PM10平均浓度控制在每立方米80微克以内。加强扬尘、油烟、机动车尾气等专项整治，完成淮化集团5台锅炉脱硫脱硝和除尘改造。加强秸秆禁烧，推进秸秆还田、发电等综合利用。实施全民节能行动计划，深化重点用能单位节能低碳行动，推广清洁生产和超洁净排放先进技术。

    全面加强水环境治理。围绕建设全国水生态文明试点市，启动国家“水十条”实施工作。加强水功能区水质监测及饮水水源地达标建设。严格控制重点行业水污染物排放，推进岸线码头及航运船舶污染整治，加快11个污水处理设施建设及运行，开展城区黑臭水体和入淮排污沟整治。推进亚行城市水系治理和瓦埠湖环境综合整治，加快焦岗湖等省级自然保护区创建。

    全面加强土壤和绿色治理。强化农业面源污染防治。启动土壤环境治理，开展土地修复试点，推进低效用地再开发和工矿废弃地复垦。推进采煤沉陷区和关闭矿区生态修复，实施一批治理项目。推进国家森林城市创建，完成成片造林3.3万亩。积极争创国家生态文明建设示范区和生态乡镇。

    （六）持续深化改革创新，不断增强发展活力

    统筹推进改革和创新工作，加快要素驱动向创新驱动转变。

    深化行政体制改革。持续推进简政放权，承接并落实省政府取消下放的审批事项，推动行政审批事项全部进中心办理，完善网上审批服务。深化商事制度改革，落实电子营业执照和全程电子化管理，简易注销程序，适时启动证照分离改革。深化财税体制改革，完善全口径政府债务管理，全面推进“营改增”扩围工作。抓好医药卫生体制综合改革试点。推进环保监测监察体制、供销社综合改革。深化城乡户籍一元化管理改革。完善招投标监管体系。推动潘集、毛集、市经开区下放权限转化利用。

    深化市场机制改革。加快建立现代企业制度，支持国有企业整体上市和兼并重组。规范政府性投融资平台建设，支持建设发展集团、产业发展集团做大做强，组建市融资担保集团。加强国有资产监管，确保国有资产保值增值。深化农村金融综合改革，推动金融机构在乡镇增设具有信贷功能的营业网点。进一步规范金融市场秩序，加强金融风险防控。

    深化创新驱动发展。制定“互联网+”行动实施方案、大数据发展三年行动计划，扩大免费无线上网覆盖面，推进中兴通讯产业园、安徽大数据交易中心、寿县“互联网+”产业园等建设。启动天使投资计划，培育一批种子期、初创期科技型企业。实施智能制造“千百十”工程，建设智慧企业100家。全年申请专利2000件。完善创新政策，促进大众创业、万众创新。做好质量强市工作，启动检验检测产业园建设，争创全国防爆电器产业知名品牌示范区。新增高新技术企业10家，高新技术产业产值占GDP比重达10%。

 （七）坚持共建共享发展，全面增进群众福祉

    顺应人民群众生活新期待，按照坚守底线、突出重点、完善制度、引导预期的要求，继续实施好33项省级民生工程，努力为群众办实事、办好事。

    实施扶贫攻坚。按照“三年集中攻坚、两年巩固提升”的要求，实施人才教育、基础设施建设、金融支持、社会帮扶四大扶贫行动计划，开展产业发展、易地搬迁、社会保障和就业助医四大脱贫攻坚工程，实施对口帮扶和“双包”活动，构建政府、市场、社会协同推进的扶贫开发格局，确保全年脱贫5万人，其中寿县3.1万人。实施“优先发展”提升行动，加快少数民族和民族聚居地区经济社会发展。

    扩大就业创业。完善创业扶持政策，鼓励以创业带动就业，建立面向人人的创业服务平台。实施更加积极的就业政策，打造众创空间等创业平台，解决结构性就业矛盾。推进大学生“工学一体”就业就学工作试点。加大就业困难群体帮扶力度，新增城镇就业4.2万人，农村劳动力转移就业3.5万人。开展就业技能培训1.9万人、创业培训4000人，加大创业担保贷款发放力度。

    优先发展教育事业。坚持把立德树人作为教育的根本任务，加快教育发展。制定促进教育健康发展考核奖惩办法。完成第二期学前教育三年行动计划。改革义务教育阶段管理体制，推进教师无校籍管理和校长职级制改革试点，建成实验中学新校区，免除高中贫困生学费，2个县区通过国家义务教育基本均衡发展县（区）认定。深化职业教育产教融合、工学结合、校企合作，促进职教集团做大做强。支持高等教育发展，建成安理大新校区。规范发展民办教育。办好特殊教育。

    完善保障体系。落实全民参保计划，实施机关事业单位养老保险制度并轨，实现行业社保市级统筹。完善城乡居民大病保险制度、城乡居民养老保险制度。继续开展工伤预防全国试点工作。完善社会救助体系，加快农村五保供养机构转型升级试点，提高城乡低保、五保供养标准，确保市社会福利保障区建成投用。构建多层次养老服务体系，加快社区日间照料中心、市级老年公寓建设，运营居家养老呼叫平台。实施好“全面两孩”政策，促进人口均衡发展。推进健康淮南建设，优化医疗卫生机构布局，提高医疗服务质量和水平，免费开放学校体育场所。建设采煤沉陷区安置房3100套，搬迁入住4300户。

    创新社会治理。落实重大决策社会稳定风险评估制度，完善社会矛盾排查预警和调处化解机制。加强和改进信访工作，有效预防和化解矛盾纠纷。严厉打击各类刑事犯罪，强化公共安全监管，不断提升群众安全感和满意度。加强安全隐患排查治理，完善安全生产监管 “五级五覆盖”责任体系。加强食品药品监管体系建设，创建省食品药品安全城市。深化社区体制改革，创建3个省级以上综合减灾示范社区。加强应急队伍建设，强化突发事件应急处置。发挥工会、共青团、妇联等群团组织作用。做好外事侨务、对台、妇女儿童和机关事务管理工作。加强人民防空、地震、气象、档案、残疾人和地方志事业发展。支持军队现代化建设，推进国防动员和后备力量建设，加快军民融合发展，争创全国双拥模范城“七连冠”。

**四、加强政府自身建设**

    实现新发展，创造新业绩，对政府自身建设提出了更高标准。我们务必按照“三严三实”要求，奋力争当“四个自觉”模范，进一步提升政府工作水平。

    坚持依法行政，规范行政行为。认真贯彻落实淮南市法治宣传教育条例，逐步将政府活动纳入法治轨道。自觉接受人大法律监督、政协民主监督、社会各界监督，提高人大代表议案建议和政协提案办理质量。强化运用法治方式化解各类矛盾纠纷，坚决维护社会稳定。启动“七五”普法，增强全社会特别是公职人员尊法学法守法用法观念。加强政府权力运行监管，投入运行“一库四平台”。制定政府规章2件。坚决执行重大行政决策法定程序，提高政府决策的科学化、民主化和法治化水平。

    坚持敢于担当，积极主动作为。始终保持奋发有为的激情、攻坚克难的毅力、善作善成的追求，推动各项工作迈上新台阶。坚持守土有责，严格落实工作责任制、项目负责制、行政问责制，确保国家和省市各项决策部署落地生根。完善绩效考核评价机制，全面整治懒政怠政，把工作落在实处、让行动走在前列。全面增强学习意识、创新意识、进取意识，不断研究新情况、解决新问题，提高引领新常态、推动新发展的专业化水平。

    坚持履职为民，提升服务效能。创新群众工作方法，完善联系服务群众机制。加强政务公开载体建设，让权力在阳光下运行。大力推广电子政务，方便群众办事。不断丰富行政服务方式，提高政府购买服务比例。坚持从具体事抓起、从身边事做起、从群众最不满意的事改起，坚决纠正损害群众利益的行为，持续巩固“三严三实”教育实践活动成果。

    坚持反腐倡廉，做到从严治政。深入推进党风廉政建设和反腐败工作，严明政治、组织、廉洁、群众、工作和生活纪律，推进制度建设全覆盖。健全廉政风险防控机制，实行权力运行痕迹管理。坚持厉行节约、反对浪费，严控“三公”经费支出。强化行政监察和审计监督，严防国有资金、资源、资产流失。严格落实“一岗双责”，坚决实行“一案双查”。严格执行廉洁自律准则、纪律处分条例等规定，做到干部清正、政府清廉、政治清明。

    各位代表！站在新起点、踏上新征程，任务艰巨、责任重大、使命光荣。让我们紧密团结在以习近平同志为核心的党中央周围，在省委省政府和市委的坚强领导下，齐心协力，开拓进取，为加快美丽淮南建设、全面建成小康社会而努力奋斗！
